# Study on the bZIP-Type Transcription Factors NapA and RsmA in the Regulation of Intracellular Reactive Species Levels and Sterigmatocystin Production of *Aspergillus nidulans*

**DOI:** 10.3390/ijms222111577

**Published:** 2021-10-27

**Authors:** Bernadett Bákány, Wen-Bing Yin, Beatrix Dienes, Tibor Nagy, Éva Leiter, Tamás Emri, Nancy P. Keller, István Pócsi

**Affiliations:** 1 Department of Molecular Biotechnology and Microbiology, Faculty of Science and Technology, University of Debrecen, H-4032 Debrecen, Hungary; bb.bakany@gmail.com (B.B.); emri.tamas@science.unideb.hu (T.E.); pocsi.istvan@science.unideb.hu (I.P.); 2 Doctoral School of Molecular Medicine, University of Debrecen, H-4010 Debrecen, Hungary; 3 Institute of Physiology, Faculty of Medicine, University of Debrecen, H-4010 Debrecen, Hungary; dienes.beatrix@med.unideb.hu; 4 State Key Laboratory of Mycology, Institute of Microbiology, Chinese Academy of Sciences, Beijing 100101, China; yinwb@im.ac.cn; 5 Department of Medical Microbiology and Immunology, University of Wisconsin, Madison, WI 53706, USA; npkeller@wisc.edu; 6 Department of Applied Chemistry, Faculty of Science and Technology, University of Debrecen, H-4032 Debrecen, Hungary; nagy.tibor@science.unideb.hu; 7 Department of Bacteriology, University of Wisconsin, Madison, WI 53706, USA

**Keywords:** *Aspergillus nidulans*, bZIP-type transcription factors, oxidative stress, secondary metabolite production, sterigmatocystin, catalase, reactive 0 species

## Abstract

Basic leucine zipper (bZIP) transcription factors play a crucial role in the environmental stress response of eukaryotes. In this work, we studied the effect of gene manipulations, including both deletions and overexpressions, of two selected bZIP transcription factors, NapA and RsmA, in the oxidative stress response and sterigmatocystin production of *Aspergillus nidulans*. We found that NapA was important in the oxidative stress response by negatively regulating intracellular reactive species production and positively regulating catalase activities, whereas RsmA slightly negatively regulated catalase activities. Concerning sterigmatocystin production, the highest concentration was measured in the *ΔrsmA* *ΔnapA* double deletion mutant, but elevated sterigmatocystin production was also found in the OE*rsmA* OE*napA* strain. Our results indicate that NapA influences sterigmatocystin production via regulating reactive species level whereas RsmA modulates toxin production independently of the redox regulation of the cells.

## 1. Introduction

bZIP-type transcription factors are widespread, conserved proteins in eukaryotes and play essential roles in the environmental stress responses of these organisms. A subgroup of bZIPs called Yap (yeast activator protein) transcription factors are well-characterized in yeasts [1]. Yap proteins are important in the establishment of resistance against reactive oxygen species (ROS) and osmotic stress. Several Yap-like proteins have been characterized in filamentous fungi with stress defense function and concomitant regulation of secondary metabolite production, e.g., AtfA, NapA, AfyapA, Aoyap1, and ApyapA in the Aspergilli [2,3,4,5,6,7,8], NcAp-1 in *Neurospora crassa* [2,9], MoAP1 in *Magnaporthe oryzae* [10,11] and PfZipA in *Pestalotiopsis fici* [12].

**Figure 1:**
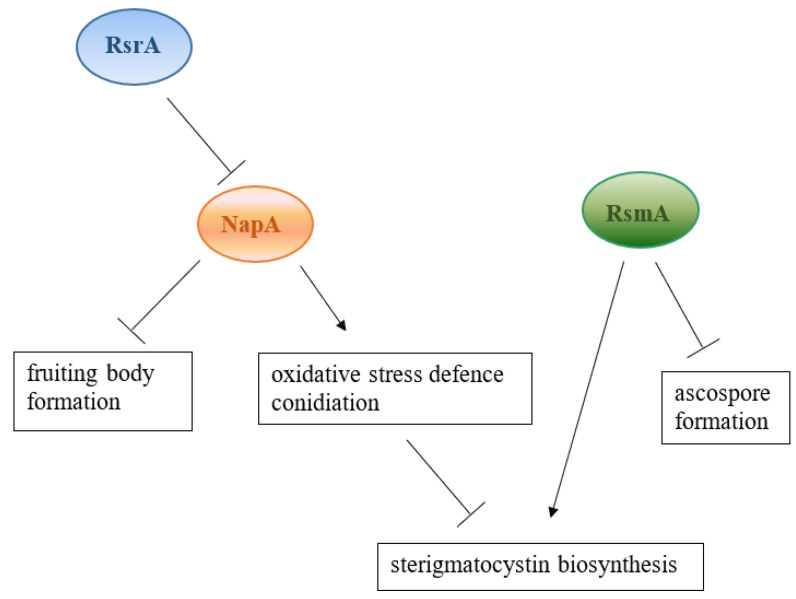
Role of NapA and RsmA in *A. nidulans.*

To study the role of NapA in *A. nidulans* both OE*napA* and *ΔnapA* mutants have been characterized (Figure 1) [13]. In the OE*napA* strain, reduced production of several secondary metabolites and imbalance in asexual/sexual development have been observed [13]. Manipulation of *napA* gene also affected the oxidative stress defense system of *A. nidulans*; namely, deletion of *napA* increased the *t*BOOH sensitivity of the fungus, with concomitantly higher reactive species (RS) production, and decreases in catalase activity in 10 h cultures and higher glutathione peroxidase activity in 24 h cultures (Figure 1) [2,13]. RS production was also higher in the OE*napA* strain compared to the wild type without disturbing the catalase and glutathione peroxidase activities [13]. In the study of Mendoza-Martínez et al. [14], it was confirmed that *napA* is induced at high ROS levels and nuclear localization of NapA is induced by H_2_O_2_, menadione and osmotic stress, glucose starvation, and growth on ethanol. NapA is also required for conidiation but represses fruiting body formation (Figure 1) [14]. NapA controls several genes involved in detoxification and drug efflux, which protect the fungus during conidiation, e.g., NapA directly activates catalase B (*catB*), the thioredoxin system, and glutathione reductase [15]. NapA is also crucial in the induction of conidiation through the oxidative stress response in the presence of redox metabolites, e.g., phenazine produced by *Pseudomonas aeruginosa* [16]. The impact of NapA on the oxidative stress defense was counterbalanced by another transcription factor, RsrA, by repressing *napA* and some NapA activated genes, such as *glrA*, *trxA*, and *catB* [17].

RsmA, (restorer of secondary metabolism A) has also been described as a Yap-like protein and is involved in the control of secondary metabolite production of *A. nidulans* [18]. Overexpression of *rsmA* restores sterigmatocystin production of *A. nidulans* in two Velvet complex deletion mutants, *ΔlaeA* and *ΔveA*, both of which greatly reduce sterigmatocystin synthesis as single deletions (Figure 1) [18]. In a previous study by Yin et al. [13], functions of RsmA in the regulation of secondary metabolism, sexual development, and stress responses were studied (Figure 1). Overexpression of *rsmA* increased sterigmatocystin production 100-fold and resulted in disturbances in ascospore formation in *A. nidulans*. RsmA activates sterigmatocystin production by binding to *aflR* (coding for a transcription factor positively regulating sterigmatocystin biosynthesis) promoter regions [19]. Interestingly, the *ΔrsmA* gene deletion strain also produced sterigmatocystin at slightly higher concentrations than the wild type, suggesting a complex regulatory role of this protein [18]. In the human pathogenic fungus *A. fumigatus*, overexpression of *rsmA* increased the concentration of twelve *gli* cluster metabolites in the culture medium and, consequently, gliotoxin production in *A. fumigatus*-infected mice. The supernatant of OE*rsmA* with higher gliotoxin concentration compared to the control inhibited human neutrophil chemotaxis in vivo [20]. Interestingly, the OE*rsmA* mutant showed growth retardation at 25 °C and increased menadione tolerance in comparison to the wild-type strain [20].

Contrarily, overexpression of the *rsmA* ortholog *AflrsmA* showed increased sensitivity to menadione in *A. flavus*, whereas deletion of *AflrsmA* resulted in menadione tolerance when compared to the wild type strain [21]. Concerning aflatoxin biosynthesis, the overexpression of *AflrsmA* increased the production of this mycotoxin as expected. Following stress treatment with menadione and *t*BOOH, aflatoxin production decreased in the *ΔAflrsmA* mutant. These observations suggest that, in *A. flavus*, AflrsmA regulates aflatoxin biosynthesis via oxidative stress signaling, although the possibility that AflrsmA can bind to *aflR* promoter regions was not examined in this work [21].

In this study, we characterized NapA and RsmA functions by construction of deletion and overexpression mutants prepared in all combinations.

## 2. Results

### 2.1. Stress Sensitivity Phenotypes of the RsmA and NapA Mutants

**Figure 2:**
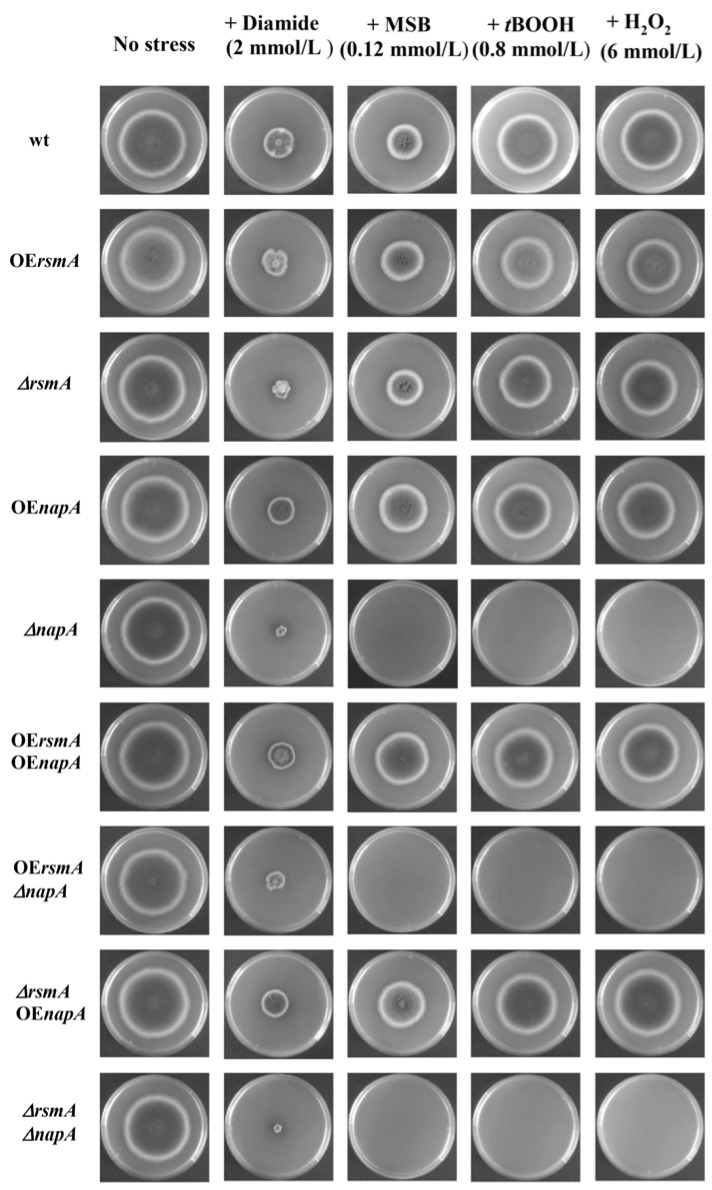
Oxidative stress sensitivity of the control and mutant *A. nidulans* strains. The oxidative stress tolerances of the mutants were tested on nutrient agar stress plates. A quantity of 10^5^ freshly grown conidia were spotted on minimal nitrate medium with 0.05 mg/L pyridoxine agar plates, which were supplemented with one of the stress0generating agents: diamide 2.0 mmol/L, MSB 0.12 mmol/L, *t*BOOH 0.8 mmol/L, H_2_O_2_ 6.0 mmol/L. The stress plates were incubated at 37 °C for 5 days.

As previously shown, deletion of *napA* yielded oxidative stress phenotypes in the presence of all tested oxidative stress generating agents, e.g., diamide, MSB, *t*BOOH, and H_2_O_2_, independently of the *rsmA* gene manipulation. Figure 2, Supplementary Table S1). The effect of *napA* on the oxidative stress-sensitive phenotype was dependent on the applied stressor, namely, in the presence of MSB decreased, and in the presence of diamide increased, the sensitivity of the mutants, whereas H_2_O_2_ sensitivity of the mutants was dependent on the *rsmA* gene. (Figure 2, Supplementary Table S1). The effect of *rsmA* deletion or overexpression on the stress sensitivity of the mutants was based on the type of the stress-generating agent and the *napA* gene (Figure 2, Supplementary Table S1).

### 2.2. Biomass, Specific RS and Specific Catalase Enzyme Productions

Deletion or overexpression of the *napA* and/or *rsmA* genes significantly affected the growth of the strains. In general, all mutant strains grew slower than the wild-type, both in untreated control and in *t*BOOH-exposed cultures (Supplementary Table S2).

**Figure 3:**
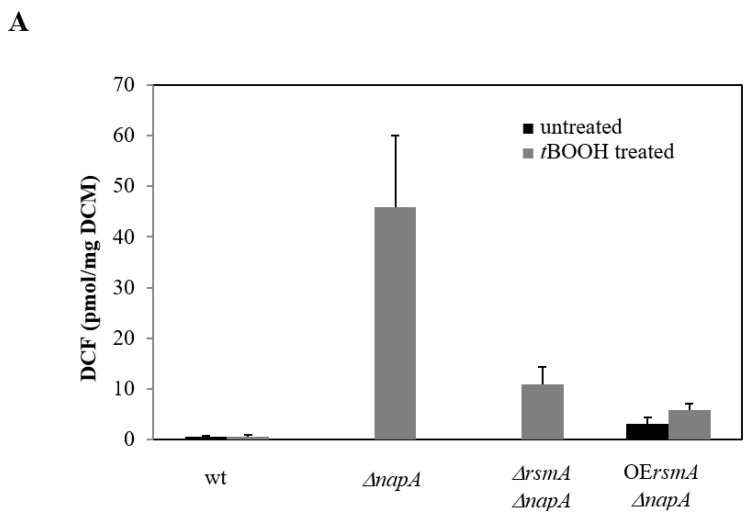

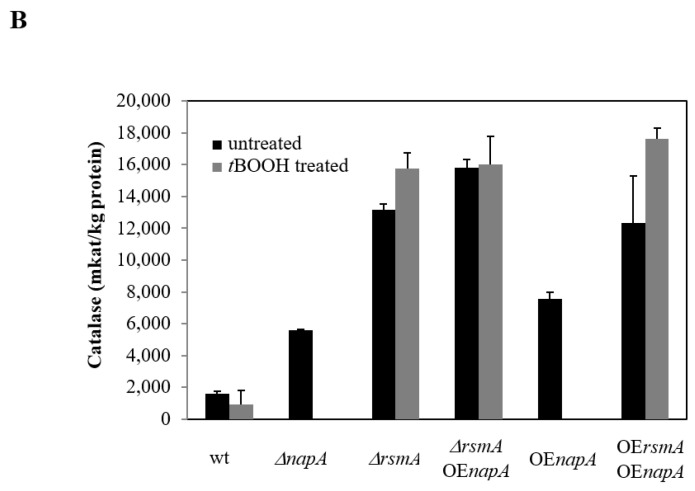
Comparison of DCF (RS) production (**A**) and catalase activities (**B**) of the mutants. Data are presented as mean ± SD values calculated from three independent experiments. Effects of the gene manipulations were analyzed by one way ANOVA followed by Tukey post-hoc test. Only data significantly different (adj. *p* < 0.05) from that of the wt strain are plotted.

In unstressed conditions, significant increases in RS production were only observed in the OE*rsmA* *ΔnapA* strain as compared to the control (Figure 3A, Supplementary Table S3). *t*BOOH treatment extensively increased the specific RS production of the *ΔnapA* mutant (Figure 3A, Supplementary Table S3). Both *rsmA* deletion and overexpression reduced RS production in the *napA* gene deletion background but were unable to re-establish the RS level of the control strain (Figure 3A, Supplementary Table S3). RS production was higher but not statistically different in the OE*rsmA* strain when compared to the wild-type strain after *t*BOOH treatment (Figure 3A, Supplementary Table S3).

Catalase production significantly increased in the *ΔnapA* mutant in comparison to the control strain without oxidative stress treatment. Neither *rsmA* overexpression nor *rsmA* deletion carried out in the *napA* gene deletion background significantly changed the catalase production measured in the *ΔnapA* strain (Figure 3B, Supplementary Table S3). Deletion of *rsmA* alone highly increased the catalase production of the fungus but RS production was similar to the control strain (Figure 3B, Supplementary Table S3). Because overexpression of *napA* alone also increased the catalase production, a remarkably high specific catalase activity was measured in the *ΔrsmA* OE*napA* strain (Figure 3B, Supplementary Table S3). The catalase activity of the *ΔrsmA* strain was comparable to that of the double overexpression mutant (Figure 3B, Supplementary Table S3). Interestingly, it was observed that *t*BOOH treatment did not influence the specific catalase activities within a given strain, and remained high in the *ΔrsmA*, *ΔrsmA*OE *napA*, and OE*rsmA* OE*napA* strains (Figure 3B, Supplementary Table S3). 

### 2.3. ST Production

**Figure 4:**
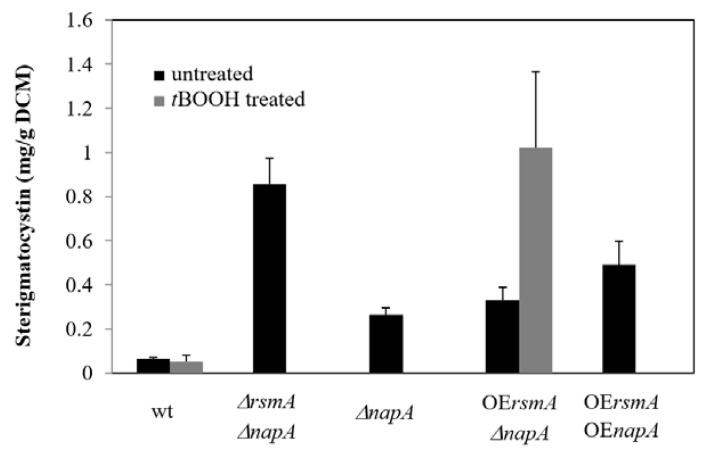
ST production of the control and mutant strains. Data are presented as mean ± SD values calculated from three independent experiments. Effects of the gene manipulations were analyzed by one way ANOVA followed by Tukey post-hoc test. Only data significantly different (adj. *p* < 0.05) from that of the wt strain are plotted.

The manipulation of *rsmA* alone did not change the sterigmatocystin production of the fungus without stress treatment (Figure 4, Table S4). The highest ST production was observed in the double deletion mutant, whereas deletion of *napA* alone also significantly increased ST production. Overexpression of *rsmA* increased the production of this toxin in the *ΔnapA* genetic background, and increased ST production was also measured in the double overexpression mutant when compared to the control (Figure 4, Table S4).

Similar to the catalase activities, no statistically significant differences in ST production were detected between the unstressed and *t*BOOH-exposed cultures within a given strain (Figure 4, Table S4). After *t*BOOH treatment, the highest ST levels were measured in the OΕ*rsmA* *ΔnapA* mutant (Figure 4, Table S4).

### 2.4. Expression Patterns

**Figure 5:**
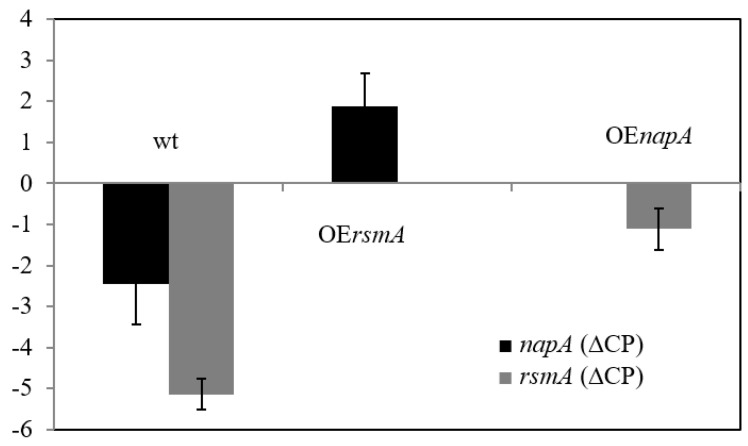
Effects of *napA* and *rsmA* overexpression on the transcriptional activity of wild type *rsmA* and *napA* genes. Data are presented as mean ± SD values calculated from three independent experiments. Effects of the gene manipulations were analyzed by one way ANOVA followed by Tukey post-hoc test.

We also examined the effect of *rsmA* on the expression of wild type *napA* and vice versa (Figure 5, Table S5). The pairwise comparison of *ΔrsmA*, control, and OE*rsmA* strains showed that OE of *rsmA* increased the transcription of *napA*, whereas OE of *napA* resulted in elevated *rsmA* expression (Figure 5, Table S5). Deletion of either *rsmA* or *napA* had no significant effect on the transcription of the other gene (Figure 5, Table S5). In the *t*BOOH-treated cultures of the control strain, the expression of *rsmA* increased in comparison to the untreated cultures (Table S5).

### 2.5. Interaction between NapA or RsmA Expressions and Specific Catalase Activities, DCF Formation, or ST Production

**Figure 6:**
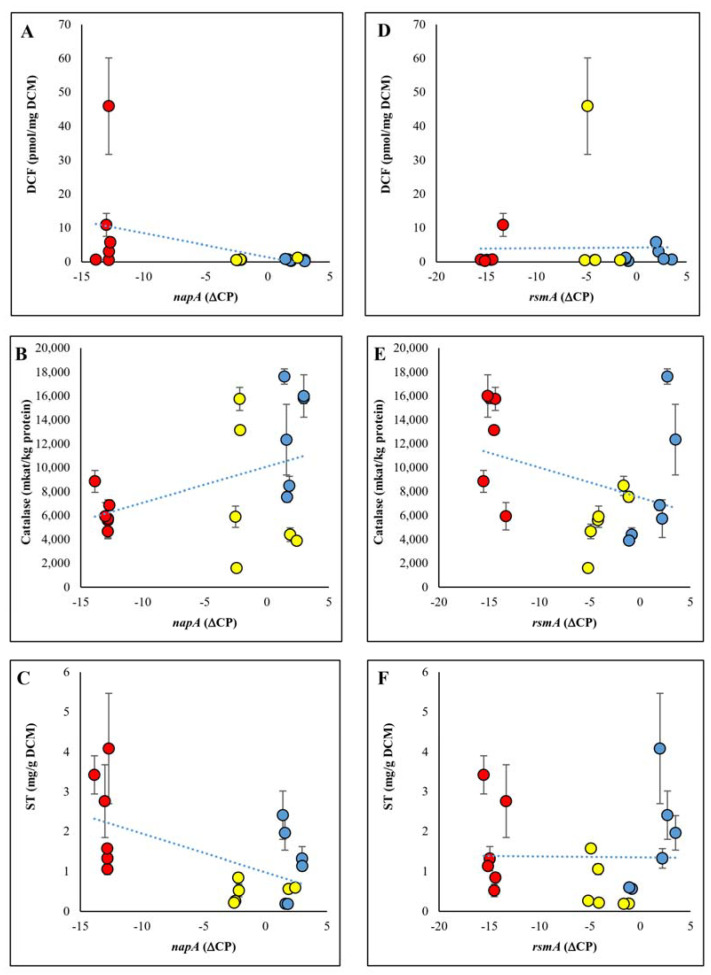
Correlation between relative transcriptions of *napA* (**A**–**C**) or *rsmA* (**D**–**F**) and DCF formations (**A**,**D**), specific catalase activities (**B**,**E**) or ST productions (**C**,**F**).

The increase in transcriptional activity of the *napA* gene tended to be associated with an increase in specific catalase activities and a decrease in RS and ST production (Figure 3, Figure 4 and Figure 6, Tables S3 and S4). In contrast, *rsmA* transcription hardly affected RS production (Figure 3 and Figure 6). Surprisingly, both increased and reduced *rsmA* transcriptions were associated with elevated catalase activities or ST production in some cultures (Figure 3, Figure 4 and Figure 6, Tables S3 and S4).

## 3. Discussion

There is strong evidence that secondary metabolite production is associated with oxidative stress, which is co-regulated by various transcription factors [7,22,23,24,25]. In this study we constructed a series of gene deletion and overexpression strains of *napA* and *rsmA*, either alone or in combination, to understand how these bZIP-type transcription factors contribute to the regulation of the stress tolerance and secondary metabolite production in *A. nidulans*.

In line with previous studies, NapA is a key player in the regulation of the oxidative stress response of *A. nidulans* [2,13,14]. Deletion of *napA* largely increased the oxidative stress sensitive phenotype of the fungus, even in the presence of pyridoxine (Figure 2), which has some antioxidant features [3]. According to gene expression studies, both *rsmA* and *napA* have an impact on each other’s expressions (Figure 5) but neither the deletion nor the overexpression of *rsmA* was unable to mitigate the absence of NapA. The increased stress sensitivity of *ΔnapA* also occurred with increased RS production but unaltered catalase activities after *t*BOOH treatment (Figure 3, Table S3).

bZIP transcription factors may also modulate secondary metabolite production in filamentous fungi via regulating ROS levels [6,7,24] or directly by binding to promoters of biosynthetic genes responsible for secondary metabolite production [8,19]. In this study we also examined the possible relationship between oxidative stress and secondary metabolite production.

The oxidative stress-sensitive *ΔnapA* mutant was characterized by increased sterigmatocystin production but, unexpectedly, the highest sterigmatocystin level was observed in the *ΔnapA ΔrsmA* double deletion mutant (Figure 4, Table S4). As reported before by Shaaban et al. [18], the deletion of *rsmA* alone may also slightly increase ST production. Previously, Yin et al. [19] reported that overexpression of *rsmA* resulted in an elevated ST production. Our data suggest that both the reduced and the elevated *rsmA* can lead to increased ST levels (Figure 4, Table S4). 

Based on these observations, we can assume that NapA regulates ST biosynthesis via modulating oxidative stress in *A. nidulans* (Figure 4, Table S4) similarly to ApyapA (*A. parasiticus*), Aoyap1 (*A. ochraceus*), and AfyapA (*A. fumigatus*) [5,7,22,23,24]. Furthermore, RsmA seems to support ST production at low intracellular RS levels [18,19] and, therefore, both oxidative stress-dependent and -independent regulatory elements are likely to modulate ST production in *A. nidulans*. Additionally, RsmA has a direct impact on ST synthesis by actively binding to and promoting AflR activity, the ST pathway specific regulator [19]. Considering the complex regulatory patterns of NapA and RsmA on the oxidative stress response and secondary metabolite production of *A. nidulans*, in addition to their impact on each other’s expressions, we can hypothesize that NapA and RsmA are likely to interact with each other either genetically or even physically to coordinate ST production (Figure 5, Table S5).

It is well known that bZIP type transcription factors may form heterodimers and coordinate a wide array of cellular processes, including the oxidative stress response, in addition to secondary metabolite biosynthesis [26]. For example, AtfA, AtfB, AtfC, and AtfD physically interact with each other in *A. fumigatus* to coordinate stress response and virulence of this opportunistic human pathogenic fungus [27]. Further research is needed to shed light on the nature of the hypothesized interaction between NapA and RsmA in *A. nidulans*.

Interestingly, *rsmA*-regulated secondary metabolite production was also stress related in *A. nidulans*, *A. fumigatus*, and *A. flavus* [20,21]. However, the *A. fumigatus* OE*rsmA* mutant was less sensitive to MSB [16] and the *A. flavus* OE*AflrsmA* strain was more sensitive to MSB [21], suggesting that the co-regulation of secondary metabolite production and oxidative stress response has species-specific components, even in fungal species belonging to the same genus. Again, this versatile regulatory pattern of bZIPs observable in the Kingdom of Fungi may be the consequence of their multilevel, easily variable, and highly flexible interactions.

## 4. Materials and Methods

### 4.1. Strains, Culture Media, and Growth Conditions

All strains are listed in Table 1. Construction of single *rsmA* and *napA* mutants are described in [13]. The four double *napA, rsmA* mutants were created by sexual crossing of single mutants according to standard methods [28]. Briefly, crossing TMS6.30 with TWY7.3 yielded RWY6.2 (*ΔrsmA∆napA*). Crossing RWY16.47 with TWY13.15 and TWY7.3 created RWY33.2 (*OE::rsmAOE::napA*) and RWY34.30 (*OE::rsmAΔnapA*), respectively. Crossing TMS6.30 with TWY13.15 yielded RWY35.5 (*ΔrsmAOE::napA*). The genotypes of the progeny were determined by growth on selection media and PCR confirmation with designated primers [13].

All strains were grown at 37 °C on Barratt’s nitrate minimal medium (NMM) supplemented with 0.05 mg/l pyridoxine [13,29].

### 4.2. Oxidative Stress Sensitivity Experiments

The oxidative stress tolerances of the mutants were tested on nutrient agar stress plates. A quantity of 10^5^ freshly grown (6 days) conidia was washed and resuspended in 0.9% NaCl, 0.01% Tween 80 [3]. Then, the conidiospores were spotted on minimal-nitrate medium agar plates, and supplemented with one of the following stress-generating agents: diamide 2.0 mmol/L (triggers glutathione redox imbalance), menadione sodium bisulphite (MSB) 0.12 mmol/L (increases intracellular superoxide concentrations), *tert*-butylhydroperoxide (*t*BOOH) 0.8 mmol/L (accelerates lipid peroxidation), or H_2_O_2_ 6.0 mmol/l (increases intracellular peroxide concentrations). All stress plates were incubated at 37 °C for 5 days [3,13].

### 4.3. Reactive Species Production and Catalase Activity

To determine the physiological parameters in submerged cultures, strains were pre-grown in Erlenmeyer flasks (500 mL) containing 100 mL minimal-nitrate medium (pH 6.5) and also supplemented with 0.05 mg/L pyridoxine. Culture media were inoculated with 10^6^ conidia/mL and incubated at 37 °C and at 3.7 Hz shaking frequency. Oxidative stress was induced by the addition of *t*BOOH (at 0.1, 0.2, and 0.4 mmol/L concentrations) to exponential growth phase (18 h) cultures. For dry cell mass (DCM) determinations, samples were taken just before the stress treatment (0 h) and at every 12 h after the stress exposure for up to 48 h. Dry cell mass (DCM) of the samples was determined as described previously [13].

The intracellular reactive species (RS) levels were characterized by the formation of 2′,7′-dichlorofluorescein (DCF) from 2′,7′-dichlorofluorescin diacetate [31]. RS includes all reactive oxygen and nitrogen species, which oxidize 2′,7′-dichlorofluorescin to DCF [31]. The amount of RS was determined at 23 h (5 h after treatment, at 0.2 mmol l^−1^ *t*BOOH). At all the incubation times tested, 2′,7′-dichlorofluorescin diacetate was added to 20 mL aliquots of the cultures at a final concentration of 30 μmol/L, and after incubating them further for 1 h in 100 mL culture flasks, mycelia were harvested by centrifugation. DCF productions were determined spectrofluorimetrically [32,33].

Changes in the specific catalase activities were also recorded in separate experiments. Submerged cultures were treated with *t*BOOH (0.2 mmol/L) at 18 h culture time. Samples were taken at 5 h after *t*BOOH treatment, and mycelia harvested by filtration were washed with distilled water and resuspended in ice-cold 0.1 M potassium phosphate buffer (pH 7.5). In these cases, cell-free extracts were prepared by disrupting mycelia with 0.5 mm glass beads (5000 rpm, 30 s) and centrifugation [32]. Catalase activities were determined spectrophotometrically, measuring H_2_O_2_ decomposition and NADPH diminution rates [34]. Protein contents of the cell-free extracts were measured by a modification of the Lowry method [35].

### 4.4. Sterigmatocystin Determination

In sterigmatocystin determinations, mycelia from 66 h cultures (48 h after stress exposure) were filtered and washed. After lyophilization, sterigmatocystin was extracted by 500 µL 70% (*v/v*) acetone from 20 mg quantities of the freeze-dried mycelial powder. Metabolites were separated in the developing solvent mixture toluene:ethyl acetate:acetic acid (TEA, 8:1:1) on silicacoated thin-layer chromatography (TLC) plates [36] and photographs were taken following exposure to UV light (λ = 366 nm).

Mycelial extracts were also analyzed by HPLC for their sterigmaticystin contents. Aliquots of 10 µL were injected into the chromatographic system, which consisted of a Waters 2695 Separations Module equipped with a thermostable autosampler (5 °C), a column module (35 °C), and a Waters 2996 photodiode array UV detector (λ = 254 nm). Separations were performed using an Agilent Zorbax SB-C18 (4.6 mm × 75 mm, 3.5 m) column with 1 mL/min flow rate. Isocratic elution was used where the mobile phase was methanol/acetonitrile/water 50/15/35 (*v/v*), respectively [13].

### 4.5. rRT-PCR Assays

Total RNA was isolated from lyophilized mycelia according to Chomczynski [37] and RT-qPCR experiments were carried out as described earlier [38]. The applied primer pairs are summarized in Table S6. Relative transcription levels were quantified with the ΔΔCP value (mean ± S.D. calculated from 4 biological replicates), which was defined as *Δ*CP_treated_ − *Δ*CP_control_, where *Δ*CP_treated_ = CP_reference gene_ − CP_tested gene_ measured in stress-treated cultures, *Δ*CP_control_= CP_reference gene_ − CP_tested gene_ measured in untreated cultures, and CP values represent the rRT-PCR cycle numbers of crossing points. As a reference gene, *actA* (AN6542) was used [38].

### 4.6. Statistical Analysis

The effects of *napA* and *rsmA* gene manipulations on the colony diameters in stress-treated and untreated surface cultures were analyzed with Dunnett’s test. The interaction between gene manipulation(s) and stress treatment was studied by two-way ANOVA.

In the case of the *t*BOOH-treated or the untreated submerged cultures, the effects of gene manipulations on catalase activity, DCF formation, and ST production, and on the relative transcription of the *rsmA* and *napA* genes, were analyzed by one way ANOVA followed by Tukey’s post-hoc test. When *t*BOOH-treated and untreated cultures were compared, the Student’s t-test with Holm’s *p*-value correction was applied. In all cases, the difference between the mean values were regarded as significant if the (adjusted) *p*-value was less than 0.05.

## Figures and Tables

**Table 1:** Strains used in this study.

Name	Genotype	Reference
RDIT 9.32	wild type	[30]
RWY 2.12	*gpdA*(*p*)::*rsmA*::*A. fumigatus pyrG*	[19]
RWY 8.5	Δ*rsmA*::*pyrG A. parasiticus*	[13]
RWY 17.3	*A. fumigatus pyroA*::*gpdA*(*p*)::*napA*, *pyroA4*	[13]
RWY 10.3	Δ*napA*::*pyroA A. fumigatus*	[13]
TWY7.3	*pyrG89*; Δ*napA*::*pyroA A. fumigatus*, *pyroA4*, Δ*nkuA*::*argB*	[13]
TMS6.30	*pyrG89; ∆rsmA::pyrG A. parasiticus, pyroA4*	[18]
TWY13.15	*pyrG89; gpdA(p)::napA:: pyroA A. fumigatus, pyroA4, ΔnkuA::argB*	[13]
RWY16.47	*AfumpyrG::gpdA(p)::rsmA, ∆aflR::argB, pyroA4, TrpC801*	[19]
RWY33.2	*gpdA(p)::napA:: pyroA A. fumigatus, AfumpyrG::gpdA(p)::rsmA* From cross of RWY16.47 X TWY13.15	This study
RWY34.30	*A. fumigatus pyrG::gpdA(p)::rsmA,∆napA:: A. fumigatus pyroA* From cross of RWY16.47 X TWY7.3	This study
RWY35.5	*ΔrsmA::A. parasiticus pyrG, A. fumigatus pyroA::gpdA(p)::napA* From cross of TMS6.30 X TWY13.15	This study
RWY6.2	*∆napA::A. fumigatus pyroA, pyroA4, pyrG89,ΔrsmA::A. parasiticus pyrG* From cross of TWY7.3 X TMS6.30	This study

All strains carry the wild type *veA* allele.

## Data Availability

Data is contained within the article and Supplementary Material.

## Supplementary Materials

The following are available online at https://www.mdpi.com/article/10.3390/ijms222111577/s1.

## References

[1] Rodrigues-Pousada C., Menezes R.A., Pimentel C. (2010). The Yap family and its role in stress response. Yeast.

[2] Asano Y., Hagiwara D., Yamashino T., Mizuno T. (2007). Characterization of the bZip-type transcription factor NapA with reference to oxidative stress response in *Aspergillus nidulans*. Biosci. Biotechnol. Biochem..

[3] Balázs A., Pócsi I., Hamari Z.S., Leiter É., Emri T., Miskei M., Oláh J., Tóth V., Hegedűs N., Prade R.A. (2010). AtfA bZIP-type transcription factor regulates oxidative and osmotic stress responses in *Aspergillus nidulans*. Mol. Genet. Genom..

[4] Hagiwara D., Asano Y., Yamashino T., Mizuno T. (2008). Characterization of bZip-type transcription factor AtfA with reference to stress responses of conidia of *Aspergillus nidulans*. Biosci. Biotechnol. Biochem..

[5] Qiao J., Kontoyiannis D.P., Calderone R., Li D., Ma Y., Wan Z., Li R., Liu W. (2008). Afyap1, encoding a bZip transcriptional factor of *Aspergillus fumigatus*, contributes to oxidative stress response but is not essential to the virulence of this pathogen in mice immunosuppressed by cyclophosphamide and triamcinolone. Med. Mycol..

[6] Reverberi M., Zjalic S., Punelli F., Ricelli A., Fabbri A.A., Fanelli C. (2007). Apyap1 affects aflatoxin biosynthesis during *Aspergillus parasiticus* growth in maize seeds. Food Addit. Contam..

[7] Reverberi M., Gazzetti K., Punelli F., Scarpari M., Zjalic S., Ricelli A., Fabbri A.A., Fanelli C. (2012). Aoyap1 regulates OTA synthesis by controlling cell redox balance in *Aspergillus ochraceus*. Appl. Microbiol. Biotechnol..

[8] Roze L.V., Chanda A., Wee J., Awad D., Linz J.E. (2011). Stress-related transcription factor AtfB integrates secondary metabolism with oxidative stress response in aspergilli. J. Biol. Chem..

[9] Tian C., Li J., Glass N.L. (2011). Exploring the bZIP transcription factor regulatory network in *Neurospora crassa*. Microbiology.

[10] Guo M., Chen Y., Du Y., Dong Y., Guo W., Zhai S., Zhang H., Dong S., Zhang Z., Wang Y. (2011). The bZIP transcription factor MoAP1 mediates the oxidative stress response and is critical for pathogenicity of the rice blast fungus *Magnaporthe oryzae*. PLoS Pathog..

[11] Tang W., Ru Y., Hong L., Zhu Q., Zuo R., Guo X., Wang J., Zhang H., Zheng X., Wang P. (2014). System-wide characterization of bZIP transcription factor proteins involved in infection-related morphogenesis of *Magnaporthe oryzae*. Environ. Microbiol..

[12] Wang X., Wu F., Liu L., Liu X., Chec Y., Keller N.P., Guo L., Yin W.-B. (2015). The bZIP transcription factor PfZipA regulates secondary metabolism and oxidative stress response in the plant endophytic fungus *Pestalotiopsis fici*. Fungal Genet. Biol..

[13] Yin W.B., Reinke A.W., Szilágyi M., Emri T., Chiang Y.M., Keating A.E., Pócsi I., Wang C.C.C., Keller N.P. (2013). bZIP transcription factors affecting secondary metabolism, sexual development and stress responses in *Aspergillus nidulans*. Microbiology.

[14] Mendoza-Martínez A.E., Lara-Rojas F., Sánchez O., Aguirre J. (2017). NapA mediates a redox regulation of the antioxidant response, carbon utilization and development in *Aspergillus nidulans*. Front. Microbiol..

[15] Thön M., Abdallah Q.A., Hortschansky P., Scharf D.H., Eisendle M., Haas H., Brakhage A.A. (2010). The CCAAT-binding complex coordinates the oxidative stress response in eukaryotes. Nucleic Acids Res..

[16] Zheng H., Kim J., Liew M., Yan J.K., Herrera O., Bok J.-W., Kelleher N.L., Keller N.P., Wang Y. (2015). Redox metabolites signal polymicrobial biofilm development via the NapA oxidative stress cascade in Aspergillus. Curr. Biol..

[17] Bok J.-W., Wiemann P., Garvey G.S., Lim F.Y., Haas B., Wortman J., Keller N.P. (2014). Illumina identification of RsrA, a conserved C2H2 transcription factor coordinating the NapA mediated oxidative stress signaling pathway in Aspergillus. BMC Genom..

[18] Shaaban M.I., Bok J.W., Lauer C., Keller N.P. (2010). Suppressor mutagenesis identifies a Velvet Complex Remediator of *Aspergillus nidulans* secondary metabolism. Eukaryot. Cell.

[19] Yin W.-B., Amaike S., Wohlbach D.J., Gasch A.P., Chiang Y.-M., Wang C.C.C., Bok J.-W., Rohlfs M., Keller N.P. (2012). An *Aspergillus nidulans* bZIP response pathway hardwired for defensive secondary metabolism operates through aflR. Mol. Microbiol..

[20] Sekonyela R., Palmer J.M., Bok J.-W., Jain S., Berthier E., Forseth R., Schroeder F., Keller N.P. (2013). RsmA regulates *Aspergillus fumigatus* gliotoxin cluster metabolites including cyclo(L-Phe-L-Ser), a potential new diagnostic marker for invasive aspergillosis. PLoS ONE.

[21] Wang X., Zha W., Liang L., Fasoyin O.E., Wu L., Wang S. (2020). The bZIP transcription factor AflRsmA regulates aflatoxin B1 biosynthesis, oxidative stress response and sclerotium formation in *Aspergillus flavus*. Toxins.

[22] Hong S.Y., Roze L.V., Linz J.E. (2013). Oxidative stress-related transcription factors in the regulation of secondarymetabolism. Toxins.

[23] Hong S.Y., Roze L.V., Wee J., Linz J.E. (2013). Evidence that a transcription factor regulatory network coordinates oxidative stress response and secondary metabolism in aspergilli. Microbiologyopen.

[24] Reverberi M., Zjalic S., Ricelli A., Punelli F., Camera E., Fabbri C., Picardo M., Fanelli C., Fabbri A.A. (2008). Modulation of antioxidant defense in *Aspergillus parasiticus* is involved in aflatoxin biosynthesis: A role for the *ApyapA* gene. Eukaryot. Cell.

[25] Reverberi M., Ricelli A., Zjalic S., Fabbri A.A., Fanelli C. (2010). Natural functions of mycotoxins and control of their biosynthesis in fungi. Appl. Microbiol. Biotechnol..

[26] Jindrich K., Degnan B.M. (2016). The diversification of the basic leucine zipper family in eukaryotes correlates with the evolution of multicellularity. BMC Evol. Biol..

[27] Silva L.P., Horta M.A.C., Goldman G.H. (2021). Genetic interactions between *Aspergillus fumigatus* basic Leucine Zipper (bZIP) transcription factors AtfA, AtfB, AtfC, and AtfD. Front. Fungal Biol..

[28] Pontecorvo G., Roper J.A., Hemmons L.M., Macdonald K.D., Bufton A.W.J. (1953). The genetics of *Aspergillus nidulans*. Advances in Genetics.

[29] Barratt R.W., Johnson G.B., Ogata W.N. (1965). Wild-type and mutant stocks of *Aspergillus nidulans*. Genetics.

[30] Tsitsigiannis D.I., Zarnowski R., Keller N.P. (2004). The lipid body protein, PpoA, coordinates sexual and asexual sporulation in *Aspergillus nidulans*. J. Biol. Chem..

[31] Halliwell B., Gutteridge J.M.C. (2007). Chapter 5. Measurement of Reactive Species. Free Radicals in Biology and Medicine.

[32] Emri T., Pócsi I., Szentirmai A. (1997). Glutathione metabolism and protection against oxidative stress caused by peroxides in *Penicillium chrysogenum*. Free Rad. Biol. Med..

[33] Emri T., Pócsi I., Szentirmai A. (1999). Analysis of the oxidative stress response of *Penicillium chrysogenum* to menadione. Free Rad. Res..

[34] Roggenkamp R., Sahm H., Wagner F. (1974). Microbial assimilation of methanol induction and function of catalase in *Candida boidinii*. FEBS Lett..

[35] Peterson G.L. (1983). Determination of total protein. Meth. Enzymol..

[36] Shwab E.K., Bok J.W., Tribus M., Galehr J., Graessle S., Keller N.P. (2007). Histone deacetylase activity regulates chemical diversity in Aspergillus. Eukaryot. Cell.

[37] Chomczynski P. (1993). A reagent for the single-step simultaneous isolation of RNA, DNA and proteins from cell and tissue samples. BioTechniques.

[38] Emri T., Szarvas V., Orosz E., Antal K., Park H., Han K.H., Yu J.-H., Pócsi I. (2015). Core oxidative stress response in *Aspergillus nidulans*. BMC Genom..

