# Supplementary material for: Study on the bZIP-Type Transcription Factors NapA and RsmA in the Regulation of Intracellular Reactive Species Levels and Sterigmatocystin Production of Aspergillus nidulans
Source: Int J Mol Sci. 2021 Oct 27;22(21):11577. doi: 10.3390/ijms222111577 (PMC8583795; doi:10.3390/ijms222111577)
Supplement: Supplementary file 1 [file ijms-22-11577-s001.zip › ijms-1426102-supplementary.pdf]

Strain		Colony diameter (mm)				
		untreated	diamide (2 mmol/l)	MSB (0.12 mmol/l)	*t*BOOH (0.8 mmol/l)	H_2O_2 (6 mmol/l)
**RWY 6.2**	***ΔrsmA ΔnapA***	60.30 ± 0.23	8.03 ± 0.85 [ab]	n.d. [ab]	n.d. [ab]	n.d. [ab]
**RWY 10.3**	***ΔnapA***	61.50 ± 0.34	10.35 ± 1.17 [ab]	n.d. [ab]	n.d. [ab]	n.d. [ab]
**RWY 34.30**	***OErsmA ΔnapA***	61.50 ± 0.57	17.93 ± 0.13 [ab]	n.d. [ab]	n.d. [ab]	n.d. [ab]
**RWY 8.5**	***ΔrsmA***	60.23 ± 1.45	13.50 ± 0.68 [ab]	32.03 ± 0.26 [a]	47.10 ± 0.85 [a]	51.98 ± 1.79 [a]
**RDIT 9.32**	**wt**	60.53 ± 0.45	28.2 ± 0.47	33 ± 0.13	50.70 ± 1.89	55.28 ± 0.57
**RWY 2.12**	***OErsmA***	58.13 ± 0.56[a]	23.98 ± 0.27 [ab]	38.19 ± 0.37 [ab]	47.78 ± 0.36 [a]	49.06 ± 0.27 [ab]
**RWY 35.5**	***ΔrsmA OEnapA***	63.98 ± 0.47 [a]	25.28 ± 0.57 [ab]	41.10 ± 0.69 [ab]	55.65 ± 0.69 [a]	60.75 ± 0.45 [ab]
**RWY 17.3**	***OEnapA***	62.55 ± 0.22[a]	26.18 ± 0.47 [ab]	43.50 ± 0.47 [ab]	51.68 ± 0.47	53.70 ± 1.11[b]
**RWY 32.30**	***OErsmA OEnapA***	63.33 ± 0.18 [a]	23.45 ± 0.27 [ab]	47.78 ± 0.37 [ab]	52.72 ± 0.26 [a]	54.21 ± 0.18[b]

Table S1 Effects of *napA* and *rsmA* gene manipulations on the colony diameters in stress treated and untreated surface cultures.
Mean±SD calculated from three independent experiments are presented.
[a] - Significant difference compared to the reference strain treated in the same way according to the Dunnatt's test ($p < 0.05$, $n = 3$).
[b] - Significant interaction between the effects of gene manipulation(s) and stress treatment on the colony diameter according to the two-way ANOVA ($p < 0.05$).
n.d. - No growth was detected.

Strain		DCM (g/l)							
		Untreated				*t*BOOH treated			
		**0 h**	**12 h**	**24 h**	**48 h**	**0 h**	**12 h**	**24 h**	**48h**
**RWY 6.2**	***ΔrsmA ΔnapA***	1.927 ± 0.24[a]	4.980 ± 0.763[a]	4.853 ± 0.411[abc]	4.013 ± 0.284[a]	1.580 ± 0.069 [ad]	4.640 ± 0.243[a]	5.587 ± 0.232[ab]	4.180 ± 0.151 [a]
**RWY 10.3**	***ΔnapA***	0.947 ± 0.133[bc]	2.460 ± 0.413[bc]	5.447 ± 0.424[ab]	3.787 ± 1.060[a]	0.827 ± 0.083[b]	1.027 ± 0.264[b]	3.953 ± 1.343[ac]	4.647 ± 0.342 [a,b]
**RWY 34.30**	***OErsmA ΔnapA***	1.050 ± 0.030[bc]	2.913 ± 0.515[bd]	5.307 ± 0.599[ab]	3.993 ± 0.810 [a]	1.240 ± 0.122[acd]	2.460 ± 0.781[cd]	5.887 ± 0.629[b]	4.387 ± 0.359 [a,b]
**RWY 8.5**	***ΔrsmA***	1.033 ± 0.023[bc]	3.900 ± 0.277[ad]	5.020 ± 0.420[abc]	3.640 ± 0.299 [a]	1.020 ± 0.171[bcd]	3.633 ± 0.512[ad]	5.727 ± 0.503[bd]	4.620 ± 0.552 [a,b]
**RDIT 9.32**	**wt**	0.747 ± 0.291[bd]	4.507 ± 0.372[a]	5.453 ± 0.348[b]	3.900 ± 0.174 [a]	0.653 ± 0.172[be]	3.233 ± 0.404[cd]	5.753 ± 0.593[bd]	4.333 ± 0.099 [a]
**RWY 2.12**	***OErmsA***	0.327 ± 0.050[d]	1.347 ± 0.261[c]	3.533 ± 0.081[de]	3.640 ± 0.382 [a]	0.340 ± 0.072[e]	0.787 ± 0.197[b]	2.080 ± 0.203[e]	4.920 ± 0.406 [a,b]
**RWY 35.5**	***ΔrsmA OEnapA***	0.627 ± 0.180[bd]	2.853 ± 0.801[bd]	3.933 ± 0.231[cdf]	4.580 ± 0.701 [a]	0.653 ± 0.031[be]	3.200 ± 0.594[cde]	4.033 ± 0.358[ac]	5.073 ± 1.210 [a,b]
**RWY 17.3**	***OEnapA***	1.260 ± 0.125[c]	3.627 ± 0.280[ab]	4.920 ± 0.203[abf]	4.567 ± 1.485 [a]	1.347 ± 0.180[ad]	3.860 ± 0.236[ae]	4.793 ± 0.194[abcd]	5.920 ± 0.733 [b]
**RWY 32.30**	***OErsmA OEnapA***	0.953 ± 0.095[bc]	2.460 ± 0.440[bc]	2.780 ± 0.579[e]	5.120 ± 0.365 [a]	0.960 ± 0.159[bc]	2.327 ± 0.376[c]	3.127 ± 0.280[ace]	4.813 ± 0.114 [a,b]

Table S2 Comparison of biomass production of the mutants.

Biomass production was characterized with DCM (g/l) values. Data are presented as mean ± SD values calculated from three independent experiments at each time point. Effects of gene manipulations were analysed by one way ANOVA followed by Tukey post-hoc test. Means marked with the same letter do not differ significantly (adj. p value < 0.05) from each other.

Strain		DCF (pmol/mg DCM)		Catalase (mkat/kg protein)	
		Untreated	*t*BOOH treated	Untreated	*t*BOOH treated
**RWY 6.2**	***ΔrsmA ΔnapA***	0.641 ± 0.227[a]	10.856 ± 3.397[a*]	8850 ± 907[a]	5937 ± 1154[acd]
**RWY 10.3**	***ΔnapA***	0.541 ± 0.244[a]	45.909 ± 14.209[b*]	5597 ± 55[ab]	4661 ± 613[acd]
**RWY 34.30**	***OErsmA ΔnapA***	3.096 ± 1.169[b]	5.751 ± 1.223[ac*]	5733 ± 1587[ab]	6857 ± 441[ad]
**RWY 8.5**	***ΔrsmA***	0.593 ± 0.181[a]	0.703 ± 0.270[c]	13142 ± 381[c]	15762 ± 971[b]
**RDIT 9.32**	**wt**	0.521 ± 0.221[a]	0.570 ± 0.216[c]	1579 ± 195[d]	897 ± 895[acd]
**RWY 2.12**	***OErsmA***	0.293 ± 0.183[a]	1.213 ± 0.434[c*]	4393 ± 569[bde]	3878 ± 202[c]
**RWY 35.5**	***ΔrsmA OEnapA***	0.615 ± 0.379[a]	0.257 ± 0.075[c]	15836 ± 473[f]	16007 ± 1779[b]
**RWY 17.3**	***OEnapA***	0.829 ± 0.269[a]	0.499 ± 0.251[c]	7555 ± 437[ae]	8474 ± 788[d]
**RWY 32.30**	***OErsmA OEnapA***	0.769 ± 0.250[a]	0.928 ± 0.276[c]	12341 ± 2967[c]	17642 ± 643[b]

Table S3 Comparison of DCF (RS) production and catalase activities of the mutants.

Data are presented as mean ± SD values calculated from three independent experiments. Effects of the gene manipulations were analyzed by one way ANOVA followed by Tukey post-hoc test. Means marked with the same letter do not differ significantly (adj. p value < 0.05) from each other. Effects of *t*BOOH treatment were studied by Student's t-test with Holm's p-value correction. Data of *t*BOOH treated cultures marked with "*" symbol significantly (adj. $p < 0.05$) differ from those of untreated cultures.

Strain		ST (mg/g DCM)	
		Untreated	*t*BOOH treated
**RWY 6.2**	***ΔrsmA ΔnapA***	0.855 ± 0.120[a]	0.690 ± 0.229[ab]
**RWY 10.3**	***ΔnapA***	0.264 ± 0.033[bc]	0.391 ± 0.023[ac]
**RWY 34.30**	***OErsmA ΔnapA***	0.330 ± 0.060[bd]	1.020 ± 0.346[b]
**RWY 8.5**	***ΔrsmA***	0.131 ± 0.039[ce]	0.211 ± 0.031[cd]
**RDIT 9.32**	**wt**	0.066 ± 0.006[e]	0.054 ± 0.026[cd]
**RWY 2.12**	***OErmsA***	0.141 ± 0.016[ce]	0.149 ± 0.023[c]
**RWY 35.5**	***ΔrsmA OEnapA***	0.329 ± 0.075[bd]	0.283 ± 0.024[ad]
**RWY 17.3**	***OEnapA***	0.048 ± 0.007[e]	0.047 ± 0.008[c]
**RWY 32.30**	***OErsmA OEnapA***	0.490 ± 0.108[d]	0.601 ± 0.152[abd]

Table S4 ST production of the control and mutant strains.
Data are presented as mean ± SD values calculated from three independent experiments. Effects of the gene manipulations were analyzed by one way ANOVA followed by Tukey post-hoc test. Means marked with the same letter do not differ significantly (adj. p value < 0.05) from each other. Effects of *t*BOOH treatment were studied by Student's t-test with Holm's *p*-value correction. No significant differences were found between the ST contents of untreated and *t*BOOH treated cultures.

Strain		Untreated		*t*BOOH treated	
		napA **(ΔCP)**	***rsmA*** **(ΔCP)**	***napA*** **(ΔCP)**	***rsmA*** **(ΔCP)**
**RWY 6.2**	***ΔrsmA ΔnapA***	-13.859 ± 1.530[a]	-15.558 ± 1.095[a]	-13.009 ± 0.978[a]	-13.328 ± 0.483[a]
**RWY 10.3**	***ΔnapA***	-12.808 ± 0.799[a]	-4.168 ± 0.350[b]	-12.818 ± 0.435[a]	-4.891 ± 0.326[b]
**RWY 34.30**	***OErsmA ΔnapA***	-12.804 ± 0.309[a]	2.211± 0.279[c]	-12.698 ± 0.100[a]	1.967 ± 0.130[c]
**RWY 8.5**	***ΔrsmA***	-2.146 ± 0.154[b]	-14.501 ± 0.838[a]	-2.191 ± 0.785[b]	-14.389 ± 1.888[ae]
**RDIT 9.32**	**wt**	-2.441 ± 0.439[b]	-5.149 ± 0.378[b]	-2.527 ± 0.642[b]	-4.108 ± 0.388[b,*]
**RWY 2.12**	***OErsmA***	1.882 ± 0.807[c]	-0.813 ± 0.469[d]	2.422 ± 0.210[c]	-1.079 ± 0.123[d]
**RWY 35.5**	***ΔrsmA OEnapA***	2.979 ± 0.257[c]	-14.956 ± 0.264[a]	2.982 ± 0.770[c]	-15.136 ± 0.200[e]
**RWY 17.3**	***OEnapA***	1.626 ± 0.527[c]	-1.109 ± 0.506[d]	1.816 ± 0.159[c]	-1.623 ± 0.212[d]
**RWY 32.30**	***OErsmA OEnapA***	1.582 ±0.455[c]	3.524 ± 0.475[c]	1.412 ± 0.368[c]	2.717 ± 0.028[c]

Table S5 Effects of *napA* and *rsmA* overexpression and gene deletion on the transcriptional activity of wild type *rsmA* and *napA* genes.
Data are presented as mean ± SD values calculated from three independent experiments. Effects of the gene manipulations were analyzed by one way ANOVA followed by Tukey post-hoc test. Means marked with the same letter do not differ significantly (adj. p value < 0.05) from each other. Effects of *t*BOOH treatment was studied by Student's t-test with Holm's p-value correction. Significant differences between the *t*BOOH treated and untreated cultures are marked with “*” symbol.

Table S6 Oligonucleotides used in this study.

**Name**	**Sequence (5'→3')**	**Purpose**
AN4562 for	TACCCCTACCCAGAACAG	forward primer of the *rsmA* gene used for rRT-PCR
AN4562rev	CCTTGGAAATCATCGCCGTG	reverse primer of the *rsmA* gene used for rRT-PCR
AN7513 for	TCTTTACCTTTCGCCTGACC	forward primer of the *napA* gene used for rRT-PCR
AN7513 rev	ACCTCGCCATTGCTGTTTG	reverse primer of the *napA* gene used for rRT-PCR
AN6542F	GAAGTCCTACGAACTGCCTGATG	forward primer of the reference gene (*actA*) used for rRT-PCR
AN6542R	AAGAACGCTGGGCTGGAA	reverse primer of the reference gene (*actA*) used for rRT-PCR
AN6700F	CCTATTCCCGAGCAAGTTC	forward primer of the reference gene used for rRT-PCR
AN6700R	TGATGTTCCTGACGATGGC	reverse primer of the reference gene used for rRT-PCR
